# Global burden of ovarian and uterine cancers attributable to high body-mass index in 204 countries and territories, 1990–2021

**DOI:** 10.3389/fonc.2025.1623926

**Published:** 2025-10-20

**Authors:** Huishan Han, Dan Hou, Qinghai Lin, Xiaohe Hao, Zhenyu Zhang, Xun Peng

**Affiliations:** ^1^ Department of Gynecology, Qilu Hospital of Shandong University, Jinan, Shandong, China; ^2^ Department of Pharmacy, Shandong Cancer Hospital and Institute, Shandong First Medical University and Shandong Academy of Medical Sciences, Jinan, China; ^3^ Department of Pathology and Lab Medicine, Shandong Cancer Hospital and Institute, Shandong First Medical University and Shandong Academy of Medical Sciences, Jinan, China

**Keywords:** ovarian cancer, uterine cancer, obesity epidemiology, global health disparities, preventive interventions

## Abstract

**Background:**

High body mass index (BMI) is a well-established risk factor for ovarian and uterine cancer. However, the global, regional, and national burden of these cancers attributable to high BMI remains underexplored. This study quantifies the trends and disparities in the burden of ovarian and uterine cancer due to high BMI from 1990 to 2021 using the Global Burden of Disease (GBD) 2021 dataset.

**Methods:**

We extracted data from GBD 2021 to estimate the mortality, incidence, and disability-adjusted life years (DALYs) attributable to high BMI for ovarian and uterine cancer across different locations and time periods. We focused on the burden of ovarian and uterine cancers among women aged 20-49. Age-standardized rates (ASRs) were calculated, and temporal trends were analyzed using the estimated annual percentage change (EAPC). Regional and national disparities were assessed using sociodemographic index (SDI) classifications. Forecasts employed the exponential smoothing (ES) and autoregressive integrated moving average (ARIMA) models.

**Results:**

Globally, the burden of ovarian and uterine cancer attributable to high BMI increased substantially from 1990 to 2021, with variations across regions and countries. High-income and upper-middle-income regions exhibited the highest ASRs, whereas low-SDI countries showed increasing trends in recent years. The EAPC analysis indicated a growing burden in developing regions, reflecting the rising prevalence of obesity. Age-stratified analysis revealed that middle-aged and older adults bear the highest burden.

**Conclusions:**

The global burden of ovarian and uterine cancer attributable to high BMI has increased significantly over the past three decades. Targeted interventions, including obesity prevention and cancer screening, are crucial for mitigating this burden, particularly in emerging high-risk regions. These findings underscore the need for urgent public health strategies to address obesity-related cancer risks worldwide.

## 1 Introduction

According to the latest GLOBOCAN 2020 estimates, ovarian and uterine cancers together constitute a substantial burden of cancer-related morbidity and mortality among women worldwide (1, 2). Ovarian cancer is the eighth most common malignancy, while uterine cancer ranks sixth.Despite advances in early detection and treatment improving outcomes for certain malignancies, the burden of ovarian and uterine cancers remains alarmingly high, particularly in high-income countries (3, 4). Ovarian cancer is often diagnosed at an advanced stage due to its subtle symptoms, making it one of the deadliest gynecological cancers with poor survival rates (5, 6). In contrast, uterine cancer is typically detected earlier due to symptomatic presentation; however, its incidence is rising in many regions, mirroring the global increase in obesity and metabolic disorders (7, 8). These epidemiological trends highlight the urgent need to identify modifiable risk factors to mitigate the growing burden of these cancers, particularly in the context of evolving demographic and lifestyle patterns.

The Global Burden of Disease (GBD) study is a systematic and scientific international collaborative project that aims to comprehensively quantify health losses caused by diseases, injuries, and risk factors at the global, regional, and national levels (9).Led by the Institute for Health Metrics and Evaluation (IHME) at the University of Washington in the United States, the project brings together thousands of researchers worldwide and represents the most comprehensive and authoritative database and research framework on population health in the world today (10).The GBD 2021 study offers detailed estimates of cancer-related mortality and disability-adjusted life years (DALYs), helping clarify the role of risk factors such as high body mass index (BMI) in cancer outcomes (11, 12). Over the last thirty years, overweight and obesity rates have climbed sharply—more than 1.9 billion adults are now overweight, and over 650 million are obese (13, 14). This global surge in excess adiposity has been implicated in numerous chronic diseases, including cardiovascular disorders, diabetes, and multiple malignancies (15, 16). Among gynecological cancers, uterine cancer exhibits the strongest association with obesity, with epidemiological studies attributing approximately 40–60% of cases to elevated BMI (17, 18). In contrast, the relationship between BMI and ovarian cancer is more complicated and varies by subtype (19). While high BMI is consistently associated with an increased risk of endometrioid and clear cell ovarian carcinomas, its role in high-grade serous ovarian cancer—the most common and lethal subtype—remains uncertain (20, 21). Still, meta-analyses indicate that each unit increase in BMI raises ovarian cancer risk by 6–10%, highlighting obesity’s significant impact at the population level (22).

The socio-demographic index (SDI), which combines income, education, and fertility rates, provides a valuable framework for examining the intersection of obesity and cancer burden (23). High-SDI countries have better healthcare but also higher rates of obesity due to sedentary lifestyles and high-calorie diets (24, 25). Paradoxically, low- and middle-SDI regions are experiencing the most rapid increases in obesity rates due to urbanization, nutritional transitions, and declining physical activity (24, 25). This shift suggests that obesity-related cancers may increasingly affect populations with limited access to cancer prevention and treatment. Although previous GBD analyses have quantified the impact of high BMI on cancers such as breast, colorectal, and pancreatic malignancies, and some studies have addressed attributable risk factors for gynecological cancers (26) a systematic assessment of the effect of high BMI on gynecological cancers—particularly ovarian and uterine cancers—remain notably scarce in the literature (27, 28).

Compounding this knowledge gap is the disruptive impact of the COVID-19 pandemic on cancer care pathways. Delays in diagnosis, surgery, and treatment for gynecological cancers may lead to more advanced stages at detection and higher mortality (29, 30). Although the long-term effects are still unknown, the pandemic has revealed how vulnerable healthcare systems are in managing chronic diseases—a challenge that will grow as obesity-related cancers increase (31). Against this backdrop, the present study aims to address three key objectives. First, it quantifies the global, regional, and national burden of ovarian and uterine cancers attributable to high BMI from 1990 to 2021 using GBD 2021 data. Second, it examines temporal trends in age-standardized mortality rates (ASMRs) and DALYs through join point regression, identifying periods of significant acceleration or deceleration. Third, it explores disparities in BMI-attributable cancer burden across SDI quintiles, geographic regions, and age groups, with a focus on women, who are primarily affected by these cancers. By integrating epidemiological, demographic, and risk factor data, this analysis aims to inform targeted prevention strategies and optimize resource allocation in alignment with global health priorities.

## 2 Method

### 2.1 Data sources

The GBD 2021 database offers comprehensive and comparable modeling of epidemiological parameters for 371 diseases across 204 countries from 1990 to 2021. It integrates diverse data sources like census files, household health surveys, vital event registration systems, consultation databases in health-care facilities, satellite remote sensing observation data, and infectious disease surveillance networks. The database uses the DisMod-MR 2.1 system, a tool based on Bayesian meta-regression algorithms, for disease burden assessment. In this study, we focused on extracting data related to the disease burden of uterine and ovarian cancers caused by high BMI, aiming to analyze the disease burden in a multifaceted manner. In particular, our research concentrated on the data concerning the disease burden of uterine and ovarian cancers among women in the 20–49 age group. The GBD risk factor hierarchy and accompanying exposure define exposure to high BMI (>25 kg/m^2^) using a theoretical minimum risk exposure level of 20–25 kg/m^2^ for BMI values (27).

### 2.2 Statistical analysis

Our temporal analysis of uterine and ovarian cancers due to high BMI began by calculating case numbers and age-standardized rates (ASRs) - covering Deaths, DALYs, YLDs and YLLs - throughout the 1990–2021 period. The formula for calculating the age-standardized rate (ASR) is as follows:


$$\begin{array}{l} \text{ASR}=(\sum_{\text{i}=1}^{\text{A}}\text{(aiwi)}/\sum_{\text{i}=1}^{\text{A}}\text{wi)}\times 100,000\;\\ \text{wi: the people number in the matching ith age group among the standard population; }\\ \text{ai: the age-specific rate in ith age group; }\\ \text{A: the number of age groups} \end{array}$$

By fitting the natural logarithm of ASR with the calendar year, y=ln (rate), x=calendar year, and *ϵ*=error term, the estimated average percentage change (EAPC) was calculated to illustrate the secular trend in ASRs of GERD burden based on a regression model. The positive or negative ASR trends are denoted by *β* in this formula. EAPC and its 95% confidence interval (CI) were computed using the formula 100×(exp (β)-1) . We examined burden variations through demographic stratification across gender, age, development levels (SDI quintiles), regional disease clusters (GBD regions), and national administrative divisions, with emphasis on 2021’s cross-sectional data. For future projections, ARIMA and ES models forecasted the trajectory of high BMI-related uterine and ovarian cancers to 2050, enhanced by ensemble methods for prediction stability. Analytical validity was maintained through uncertainty quantification (95% UI) with *p* < 0.05 thresholds, implemented in R version 4.2.2.

## 3 Results

### 3.1 Temporal trend for GBD of uterine cancer or ovarian cancer due to high BMI from 1990 to 2021

**Figure 1:**
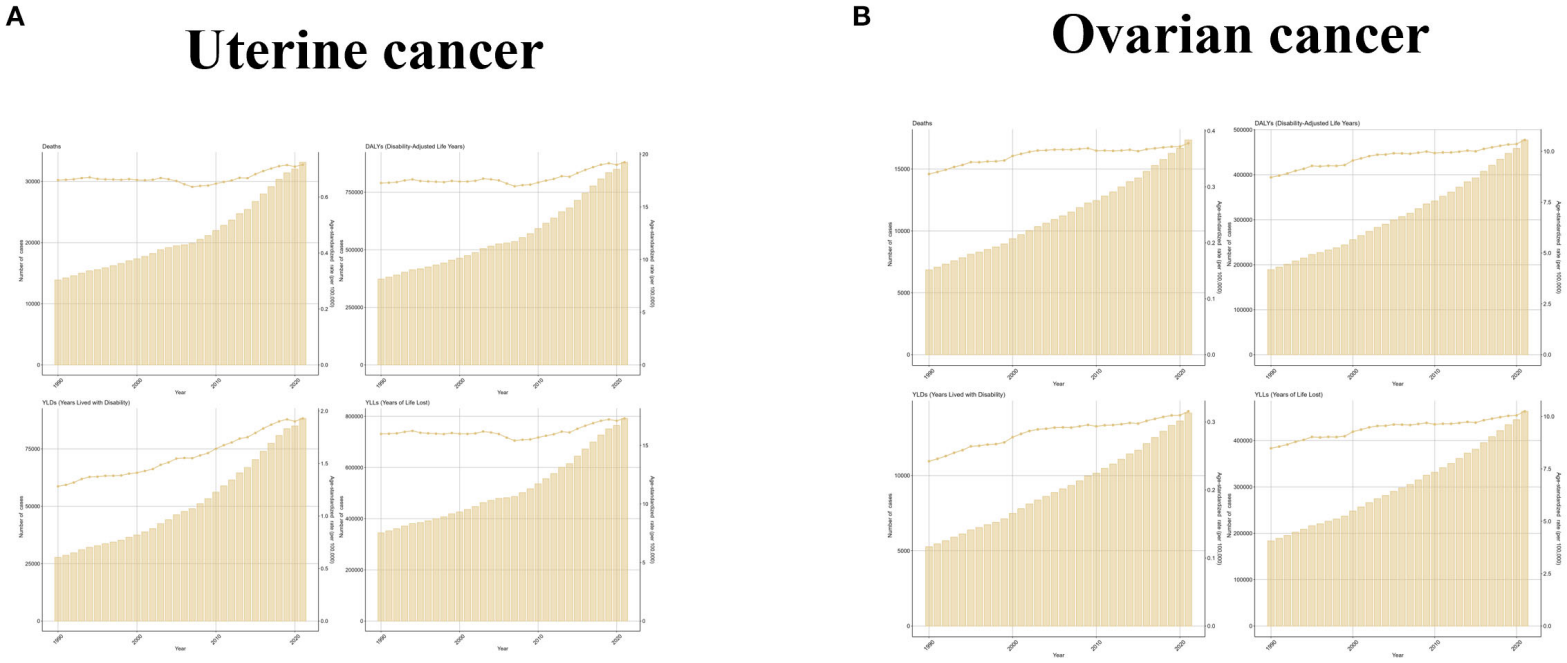
The trend of uterine cancer or ovarian cancer due to high BMI-related GBD of deaths, YLDs, YLLs and DALYs between 1990 and 2021. ASR, age-standardized rate; YLDs, Years Lived with Disability; YLLs, Years of Life Lost; DALYs, disability-adjusted-life-years.

Globally, the number of cases of uterine cancer due to high BMI has shown a steady upward trend over time, with DALYs, Deaths, YLDs, and YLLs increasing from 372641 (95% UI: 264224-500197), 13893 (95% UI: 9874-18653), 27755 (95% UI (95%UI: 17250-40423) and 344887 (95%UI: 243486-461586) in 1990 to 880147 (95%UI: 631165-1160930), 33134 (95%UI: 23878-43299), 88263 (95%UI: 56066-125024), and 791884 (95%UI: 791884). The ASRs of the other indicators fluctuated slightly before 2007 and rose after 2007, except for the ASR of YLDs, which showed an upward trend, with EAPCs of 0.86 (95%UI: 0.68 to 1.05), 0.82 (95%UI: 0.62 to 1.03), 1.97 (95% UI: 1.79 to 2.15) and 0.76 (95% UI: 0.57 to 0.94) for DALYs, Deaths, YLDs, and YLLs, respectively. The number of cases of ovarian cancer due to high BMI also showed an upward trend over time. Their DALYs, Deaths, YLDs, and YLLs increased from 1888874 (95% UI: 38401-355691), 6850 (95% UI: 1423-12865), 5269 (95% UI: 1012-10441), and 183605 (95% UI: 37368-345920) in 1990 growing to 477248 (95% UI: 113449-840002), 17344 (95% UI: 4141-30810), 14149 (95% UI: 3284-26704), and 463099 (95% UI: 110442-815411) in 2021. Each ASR has an overall slight upward trend except for a downward change between 2009–2010 and 2014-2015. The EAPCs of ASR for DALYs, Deaths, YLDs and YLLs were 1.09 (95% UI: 0.93 to 1.25), 1.03 (95% UI: 0.85 to 1.21), 1.3 (95% UI: 1.14 to 1.45) and 1.08 (95% UI: 0.92 to 1.24), respectively (**Figure 1** and **Table 1**).

**Table 1:** The change of uterine cancer or ovarian cancer due to high BMI-related GBD of deaths, YLDs, YLLs and DALYs globally between 1990 and 2021.

Disease	Indicators	Number in 1990	ASR in 1990	Number in 2021	ASR in 2021	EAPC
uterine cancer	DALYs	372641 (264224-500197)	17.26 (12.25-23.16)	880147 (631165-1160930)	19.23 (13.8-25.38)	0.86 (0.68-1.05)
	Deaths	13893 (9874-18653)	0.66 (0.47-0.89)	33134 (23878-43299)	0.72 (0.52-0.94)	0.82 (0.62-1.03)
	YLDs	27755 (17250-40423)	1.28 (0.8-1.87)	88263 (56066-125024)	1.93 (1.22-2.73)	1.97 (1.79-2.15)
	YLLs	344887 (243486-461586)	15.98 (11.29-21.39)	791884 (567897-1041295)	17.31 (12.4-22.75)	0.76 (0.57-0.94)
ovarian cancer	DALYs	188874 (38401-355691)	8.72 (1.78-16.41)	477248 (113449-840002)	10.56 (2.5-18.57)	1.09 (0.93-1.25)
	Deaths	6850 (1423-12865)	0.32 (0.07-0.61)	17344 (4141-30810)	0.38 (0.09-0.67)	1.03 (0.85-1.21)
	YLDs	5269 (1012-10441)	0.24 (0.05-0.48)	14149 (3284-26704)	0.32 (0.07-0.6)	1.3 (1.14-1.45)
	YLLs	183605 (37368-345920)	8.48 (1.73-15.96)	463099 (110442-815411)	10.24 (2.44-18.02)	1.08 (0.92-1.24)

ASR, age-standardized rate; YLDs, Years Lived with Disability; YLLs, Years of Life Lost; DALYs, disability-adjusted-life-years.

**Figure 2:**
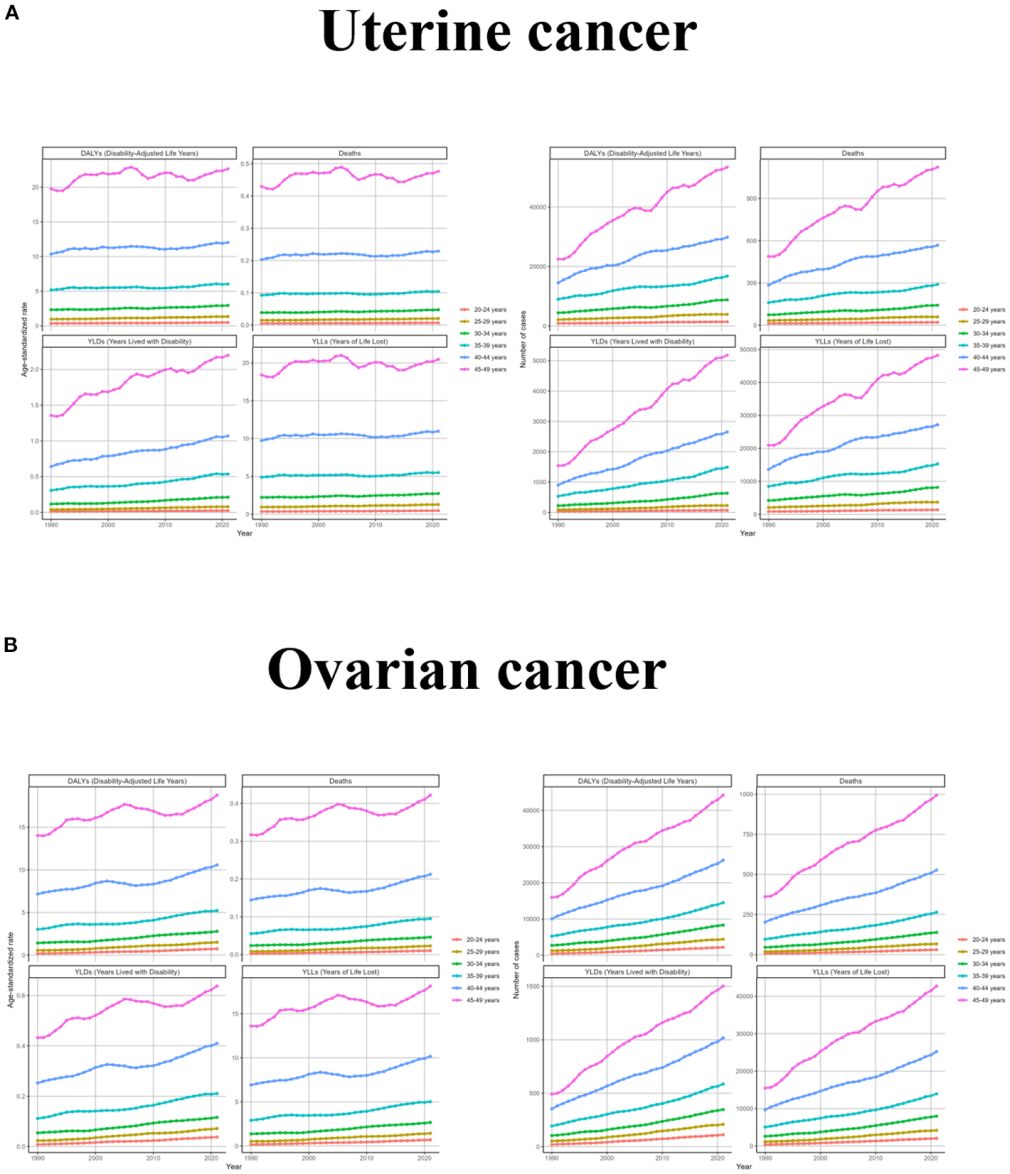
The trend of uterine cancer or ovarian cancer due to high BMI-related GBD of deaths, YLDs, YLLs and DALYs between for different age groups between 1990 and 2021. ASR, age-standardized rate; YLDs, Years Lived with Disability; YLLs, Years of Life Lost; DALYs, disability-adjusted-life-years.

Since the diseases studied were uterine and ovarian cancers, which occur only in women, and since the target population of this study was women of childbearing age, the burden of disease was analyzed only in women aged 20–49 years. From the results, it was observed that the ASR levels of uterine cancer due to high BMI were low and not very variable in all the age groups 20-34, and the trend in the age group 45–49 was consistent with the global trend and had the highest levels; the number of cases in all the age groups was also consistent with the global trend, and the slopes of the growth curves were proportional to the age groups. Changes in ovarian cancer due to high BMI by age group were characterized similarly to uterine cancer due to high BMI, with women under 39 years of age experiencing smaller changes and a high degree of concordance with global trends. In contrast, changes in the 45–49 age group were more volatile, experiencing a decade-long downward trend between 2005 and 2015. Changes in the number of cases were consistent with uterine cancer (**Figure 2** and **Supplementary Table S1**).

**Figure 3:**
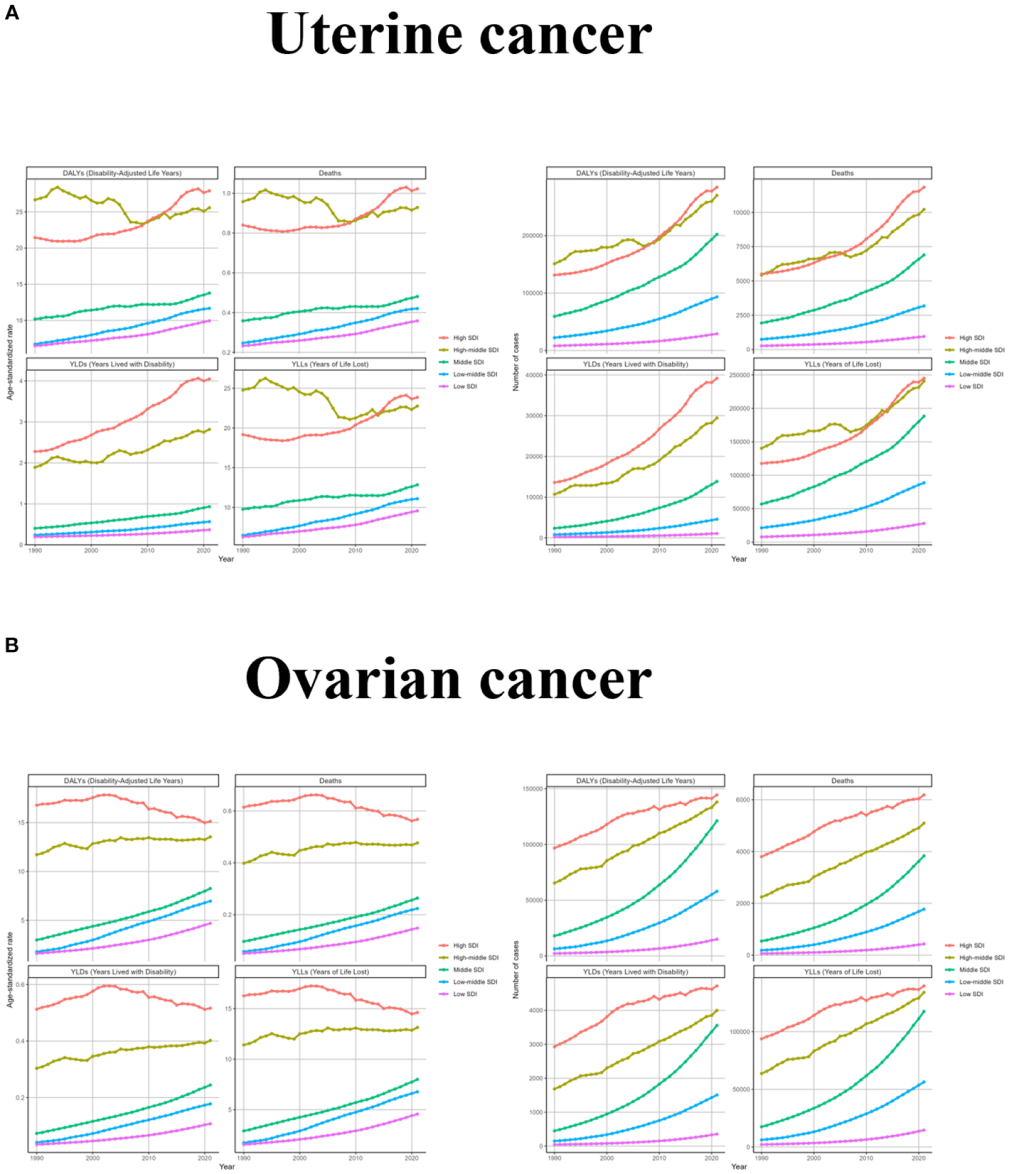
The trend of uterine cancer or ovarian cancer due to high BMI-related GBD of deaths, YLDs, YLLs and DALYs between for different SDI regions between 1990 and 2021. ASR, age-standardized rate; YLDs, Years Lived with Disability; YLLs, Years of Life Lost; DALYs, disability-adjusted-life-years.

Changes in different SDI regions varied significantly depending on the development of each region. The changes in ASR for uterine cancer due to high BMI were more consistent in High SDI, Middle SDI, Low-middle SDI, and Low SDI regions, showing a rise with increasing years, while the High- middle SDI region had a more zigzagging trend, with a fluctuating decline until 2007 and a zigzagging rise thereafter. Changes in the number of cases were consistent with the global trend, except for the High- middle SDI region, which had fluctuating year intervals in the middle. For ovarian cancer due to high BMI, ASR increased steadily over time in Middle SDI, Low-middle SDI, and Low SDI regions, fluctuated but rose slightly in High- middle SDI region, and only High SDI region showed a downward trend after 2002. From a caseload perspective, all regions have generally increased, with the slope of increase being greatest in Low-middle SDI and smallest in Low SDI (**Figure 3** and **Supplementary Table S2**).

**Figure 4:**
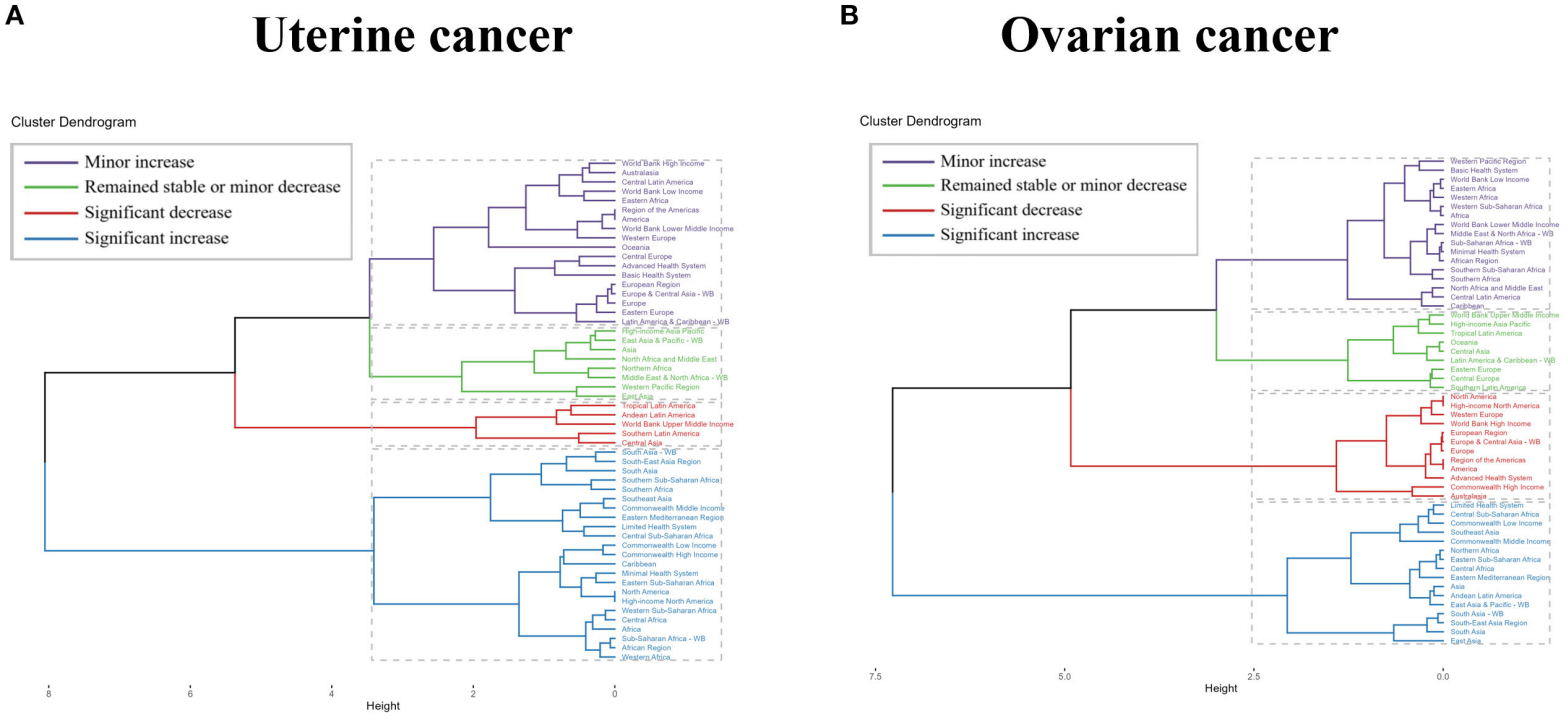
Results of cluster analysis based on the EAPC values of uterine cancer or ovarian cancer due to high BMI-related age-standardized rates for deaths and DALYs from 1990 to 2021. EAPC, estimated annual percentage change; DALYs, disability-adjusted-life-years.

To explore changes in burden across GBD regions, we performed a hierarchical cluster analysis of ASRs for all indicators, as shown in **Figure 4**. For uterine cancer due to high BMI, there were only five regions with significant decreases, including Tropical Latin America, Andean Latin America, World Bank Upper Middle Income, Southern Latin America, and Central Asia. There are many more significant increases in South Asia - WB, South-East Asia Region, South Asia, Southern Sub-Saharan Africa, Southern Africa, Southeast Asia, Commonwealth, Middle Income, Eastern Mediterranean, and South Asia. Middle Income, Eastern Mediterranean Region, Limited Health System, Central Sub-Saharan Africa, Commonwealth Low Income, Commonwealth High Income. Caribbean, Minimal Health System, Eastern Sub-Saharan Africa, North America, High-income North America, Western Sub-Saharan Africa, Central Africa, Sub-Saharan Africa -WB, African Region and Western Africa. For uterine cancer due to high BMI, there were 12 regions with significant decreases, including North America, High-income North America, Western Europe, World Bank High Income, European Region, Europe & Central Asia - WB, Europe, Region of the Americas, America, Advanced Health System, Commonwealth High Income, and Australasia. There were many more significant increases, including Limited Health System, Central Sub-Saharan Africa, Commonwealth Low Income, Southeast Asia, Commonwealth Middle Income, Northern Africa, Eastern Sub- Saharan Africa, Central Africa, Saharan Africa and Australasia. Saharan Africa, Central Africa, Eastern Mediterranean Region, Asia, Andean Latin America, East Asia & Pacific - WB, South Asia - WB, South-East Asia Region, South Asia and East Asia (**Figure 4** and **Supplementary Table S3**).

**Figure 5:**
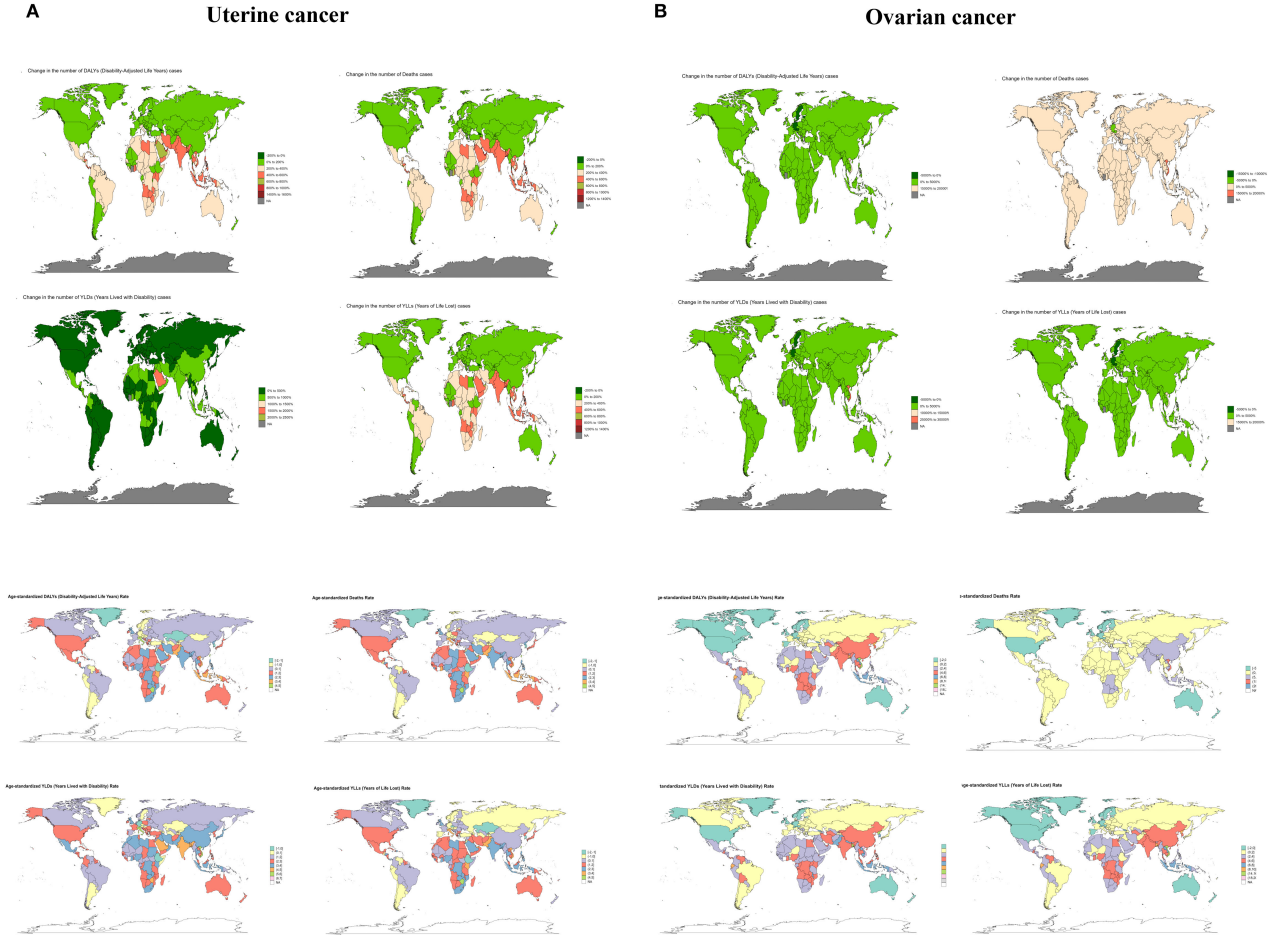
The trend of uterine cancer or ovarian cancer due to high BMI-related numbers and ASRs of deaths, YLDs, YLLs and DALYs between for different countries between 1990 and 2021. ASR, age-standardized rate; YLDs, Years Lived with Disability; YLLs, Years of Life Lost; DALYs, disability-adjusted-life-years.

As the burden of disease varies across countries due to economic and policy changes over time, it is useful to trace the history of countries with large burden reductions in order to improve reference for the development of disease prevention and control measures. For uterine cancer caused by high BMI, Taiwan (Province of China) had the greatest increase in burden, with EAPC values of 5.96 (5.46 to 6.47), 5.78 (5.26 to 6.3), 7.44 (6.91 to 7.98), and 5.79 (5.28-6.29) for DALYs, Deaths, YLDs, and YLLs, respectively. Except for YLDs (EAPC=0.01, 95%UI: -1.24 to 1.27), which were the least burdensome (all countries had positive YLDs), Ethiopia had DALYs (EAPC=-1.4, 95%UI: -2.63 to -0.16), Deaths (EAPC=-1.12, 95%UI: -2.5 to 0.28) and YLLs (EAPC=-1.44, 95%UI: -2.67 to -0.2) were the most reduced burdens. For ovarian cancer due to high BMI, the country with the largest increase in burden was Viet Nam, with all EAPC values > 10. The countries with the largest decreases in burden were all Sweden, with EAPC values for DALYs, Deaths, YLDs and YLLs of -1.09 (95%UI: -1.79 to -0.38), -0.73 (95%UI: -1.61 to 0.17), -1.11 (95%UI: -1.74 to -0.48) and -1.09 (95%UI: -1.79 to -0.38), respectively (**Figure 5** and **Supplementary Table S3**).

### 3.2 The disease burden of uterine cancer or ovarian cancer due to high BMI in 2021

In order to visualize the burden of uterine cancer or ovarian cancer due to high BMI in recent times, we have elaborated on the burden in 2021. The global burden in 2021 has been described above, and next we analyze it in terms of different age groups, SDI regions, GBD regions, and countries.

Among women of childbearing age, it can be visualized that the number of cases and ASR for DALYs, Deaths, YLDs, and YLLs are positively correlated with age, regardless of whether it is uterine cancer or ovarian cancer due to high BMI, and the values converge to zero in the 20–29 age group, with the burden in the 45–49 age group were the most burdensome, with specific values shown in **Supplementary Table S1** (**Supplementary Figure S1** and **Supplementary Table S1**).

Similar to the trend for age, the burden of each SDI region was positively proportional to the SDI level, as shown by High SDI> High-middle SDI> Middle SDI> Low-middle SDI> Low SDI, in both uterine and ovarian cancers due to high BMI. The DALYs, Deaths, YLDs and YLLs of uterine cancer in the High SDI region were 284156 (95%UI: 206127-368034), 11838 (95%UI: 8412-15583), 39181 (95%UI: 25137-56020) and 244975 (95%UI: 177672-318124), with ASR of 27.91 (95%UI: 20.5-36.15), 1.02 (95%UI: 0.74-1.34), 4.05 (95%UI: 2.57-5.79) and 23.86 (95%UI: 17.53-30.88) respectively. The number of cases of DALYs, Deaths, YLDs and YLLs of ovarian cancer in Low SDI region were 14943 (95%UI: 2349-28748), 433 (95%UI: 68-840), 355 (95%UI: 55-710) and 14588 (95%UI: 2294-28056) with ASR of 4.68 (95%UI: 0.74-9.04), 0.15 (95%UI: 0.02-0.29), 0.11 (95%UI: 0.02-0.22) and 4.57 (95%UI: 0.72-8.85), respectively (**Supplementary Figure S2** and **Supplementary Table S2**).

Significant differences occur between different regions due to uneven development of social, economic and medical levels. For uterine cancer due to high BMI, except for YLDs, the maximum value of ASR was fixed in High-income North America (7.42, 95%UI: 4.76-10.5) and North America (7.42, 95%UI: 4.76-10.5), the rest of the maximums were encompassed by Eastern Europe, with 60.22 (95%UI: 42.49-79.04), 2.1 (95%UI: 1.47-2.75), and 53.36 (37.28-70.27) for DALYs, Deaths, and YLLs, respectively. South Asia occupied the minimum. The largest number of cases of uterine cancer due to high BMI were in the Advanced Health System, with DALYs of 475080 (95%UI: 342,833-615,426). Deaths of 19359 (95% UI: 13740-25369), YLDs of 61469 (95% UI: 40045-88086), and YLLs of 413611 (95% UI: 297935-537225). While Oceania had all the smallest values. For ovarian cancer due to high BMI, the ASR maxima and minima were predominantly located in Eastern Europe and High-income Asia Pacific, whereas the maxima of number of cases, consistent with uterine cancer, were localized in the Advanced Health System, whose DALYs, Deaths, YLDs, and YLLs were 232787 (95% UI: 58637-405205), 9605 (95% UI: 2406-16919), 7235 (95% UI: 1857-13556), and 225551 (95% UI: 57009-393775), respectively (**Supplementary Figure S3** and **Supplementary Table S3**).

Differences were analyzed from the administrative country perspective. uterine cancer due to high BMI, United Arab Emirates had the largest ASR values for all of them, which were 112.77 (95%UI: 73.91-170.03), 5.35 (95%UI: 3.45-8.09), 8.92 (95%UI: 5.47-14.13) and 103.85 (95%UI: 67.73-155.94) for DALYs, Deaths, YLDs and YLLs. While the minimum values were scattered in Viet Nam and Nepal. In terms of number of cases, United States of America are all the largest with 136850 (95%UI: 99557-171971), 5242 (95%UI: 3707-6683), 22397 (95%UI: 14367-31515) and 114453 (95%UI: 83173- 144011) for DALYs, Deaths, YLDs and YLLs. The largest country with ASR for ovarian cancer due to high BMI was United Arab Emirates as with uterine cancer. while the smallest value was positioned in Burkina Faso with 0.82 (95%UI: -0.15-2.21), 0.02 (95%UI: -0.01-0.06), 0.02 (95%UI: 0-0.05) and 0.8 (95%UI: -0.15-2.16) for DALYs, Deaths, YLDs and YLLs. In addition, the United States of America also had the highest burden number for Ovarian cancer, consistent with uterine cancer (**Supplementary Figure S4** and **Supplementary Table S4**).

### 3.3 The predicted results of disease burden for uterine cancer or ovarian cancer due to high BMI from 2022 to 2050

**Figure 6:**
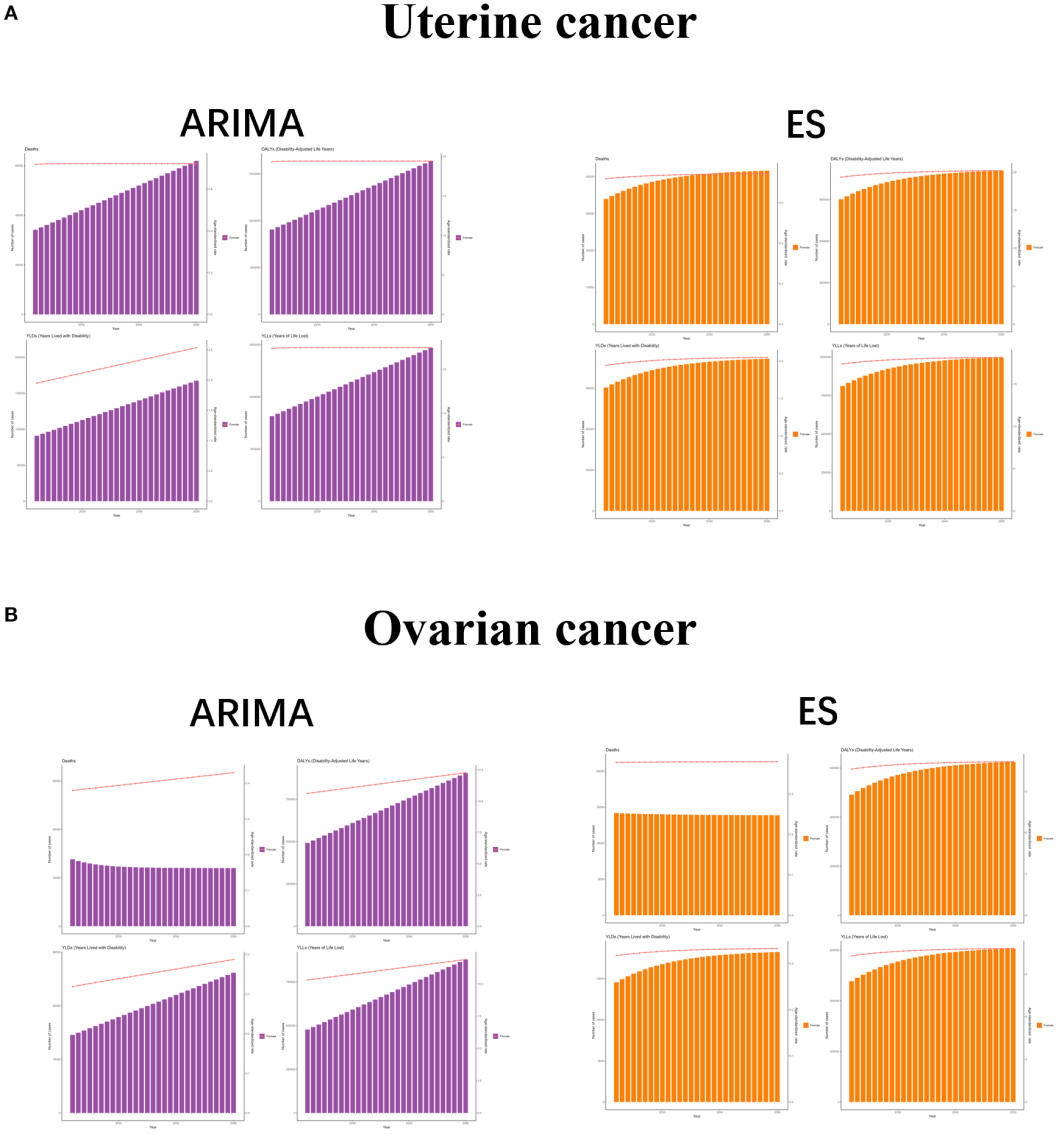
The predicted results in uterine cancer or ovarian cancer due to high BMI -related GBD of deaths, YLDs, YLLs and DALYs from 2022 to 2050 by ARIMA and ES model. ASR, age-standardized rate; YLDs, Years Lived with Disability; YLLs, Years of Life Lost; DALYs, disability-adjusted-life-years.

In order to better formulate prevention and control policies for uterine cancer or ovarian cancer due to high BMI, we used ARIMA and ES models to predict the future burden. Both ARIMA and ES models showed that the number of cases of each indicator of uterine cancer due to high BMI increased over time, while the ASR showed a constant or small upward trend, while ASR showed a constant or slight upward trend. For ovarian cancer due to high BMI, the ARIMA results showed an increase in all indicators except for the number of deaths, and the ES model showed an increase in all indicators except for the number and the ASR of deaths, which were stable or even trending downward. These results suggest that the burden of uterine cancer or ovarian cancer due to high BMI is still increasing, and there is an urgent need to develop effective measures to mitigate its impact on human health (**Figure 6**).

## 4 Discussion

Our analysis provides critical insights into the escalating burden of uterine and ovarian cancers attributable to high BMI across diverse populations, geographic regions, and socioeconomic strata. Globally, the burden of these cancers has increased substantially over time, driven by a steady rise in case numbers. ASR for DALYs, Deaths, YLDs and YLLs. The ASRs for uterine cancer related to high BMI showed consistent increases across all SDI regions except for high SDI. In 2021, the disease burden remained strongly age-dependent, with the highest DALYs, deaths, YLDs, and YLLs observed in women aged 45–49. Model-based projections suggest this trend will continue. Globally, ASR for DALYs of uterine cancer cases attributable to high BMI surged from 17.26 in 1990 to 17.26 in 2021, accompanied by parallel increases in ASR for YLDs, and YLLs. ASR for (32, 33). The increase in DALYs over three decades underscores the compounding effects of population aging, rising obesity prevalence, and prolonged exposure to adiposity-related carcinogenic pathways (34). Rising ASRs for both cancers indicate limited progress in obesity prevention and underscore the long delay between high BMI exposure and cancer development (18, 35). Although absolute case numbers have grown due to demographic changes, stable ASRs in some high-SDI regions since 2002 suggest local successes in obesity control. Still, global ASRs for uterine and ovarian cancers continue to rise annually by 1.65% and 1.32%, respectively. Age-specific patterns were also observed. Uterine cancer was most common in women aged 45–49, likely due to hormonal and metabolic changes around menopause. Younger women (20–34 years) had few cases, possibly because of less obesity exposure and protective hormonal factors (36). Furthermore, the steepest increase in case numbers occurred in older age groups, reflecting both population aging and prolonged exposure to obesogenic risk factors. Ovarian cancer showed similar age trends, with the highest burden in the 45–49 age group. The transient decline in case number between 2005 and 2015 for this age group may reflect improvements in early detection or temporary fluctuations in obesity prevalence, though further investigation is warranted. Notably, the rate of case number growth correlated positively with age, underscoring the cumulative carcinogenic impact of chronic obesity.

SDI regions exhibited distinct trajectories in the burden of uterine cancer. High- and middle-SDI regions experienced steady increases in ASRs, whereas high-middle-SDI areas showed greater variability, with a nadir in 2007 followed by irregular fluctuations. This may reflect economic changes, including urbanization and shifts to Western diets, which disrupted traditional lifestyles. Low and low-middle-SDI regions had sustained ASR growth, likely due to weak health systems and slow adoption of obesity prevention programs (37). Conversely, high-SDI regions experienced a decline in ASRs after 2002, attributed to robust public health interventions—such as sugar taxes, fitness initiatives, and early cancer screening programs. Although case numbers rose in all SDI regions, the biggest increases were in low-middle-SDI areas, partly due to population growth and shifts toward high-calorie diets. These patterns highlight the challenge of managing obesity-related cancer risk in developing economies while maintaining progress in wealthier nations. We also noticed the geographic disparities were stark. For uterine cancer, high-income North America and Eastern Europe dominated ASR rankings, driven by obesity prevalence and aging populations. Conversely, Australasia reported the lowest ASRs, possibly due to culturally embedded active lifestyles and stringent food labeling policies. For ovarian cancer, Eastern Europe and high-income Asia Pacific had the highest ASRs, regions with high rates of metabolic syndrome and genetic risks (e.g., BRCA mutations) (38, 39). At the national level, the United Arab Emirates (UAE) led in ASRs for both cancers, reflecting rapid urbanization, sedentary behaviors, and a 40% adult obesity rate. Conversely, Burkina Faso and Nepal reported minimal burdens, consistent with lower obesity prevalence and competing mortality risks (e.g., infectious diseases). The United States accounted for the highest absolute cases (136,850 uterine; 52,420 ovarian), underscoring its large population and pervasive obesogenic environment.

Obesity contributes to gynecological cancers through multiple biological mechanisms. Fat tissue dysfunction leads to chronic inflammation, insulin resistance, and high estrogen levels—all of which can promote cancer (40, 41). In uterine cancer, excess fat increases estrogen production and lowers sex hormone-binding globulin (SHBG), leading to endometrial overgrowth and higher cancer risk (42–45). For ovarian cancer, metabolic changes such as high insulin and leptin levels may trigger cancer through pathways like PI3K/AKT/mTOR and weaken immune responses (41, 42). Additionally, adipokines such as adiponectin and leptin play key roles in modulating cellular proliferation, angiogenesis, and metastasis in both cancer types (46). These mechanisms highlight the important role of obesity in uterine and ovarian cancers.

Looking ahead, both ARIMA and ES models predict continued growth in uterine cancer cases, with annual increases of 2.3% to 3.1%. ASRs may stabilize or rise slightly (EAPC: 0.8%–1.2%), balancing better obesity control against aging populations. High SDI regions may experience declining ASRs due to targeted interventions, whereas low SDI regions face a concerning trajectory, with cases potentially doubling by 2040. Model differences reflect uncertainties: ARIMA predicts rising ASRs for most measures, while ES suggests stable or declining ASRs. This may be due to better early detection reducing death rates, while rising obesity drives new cases. By 2050, the global DALYs for ovarian cancer attributable to high BMI could surpass 120,000, with the highest burden projected in Eastern Europe and North America. Policymakers in these regions should prioritize actions to reduce obesity-related cancers and their costs.

Several limitations should be considered when interpreting our findings. First, BMI data come from surveys and exams, which may include errors and misclassify individuals (47). Additionally, while obesity is a known risk factor, different obesity phenotypes may have varying associations with the risk of these cancers (48). However, GBD 2021 did not account for these distinctions, which could influence the observed trends. Furthermore, data quality varies by region, especially in low-SDI countries where underreporting and limited healthcare may underestimate the true burden. The lack of detailed information on histological subtypes and anatomical locations of uterine and ovarian cancers also constrained our ability to conduct a more nuanced analysis. Moreover, although we used the latest data, the COVID-19 pandemic’s impact on cancer trends is not included. Finally, the ecological design of this study presents inherent limitations, as it does not establish causality at an individual level. Future research should focus on analytical longitudinal studies incorporating individual-level data and additional risk factors, such as hormonal influences, metabolic conditions, and lifestyle factors, to further elucidate the complex relationship between high BMI and the burden of uterine and ovarian cancers.

## 5 Conclusion

The GBD 2021 findings highlight the growing burden of high BMI-related uterine and ovarian cancers, particularly among aging populations and economies in transition. While some progress has been observed in high-SDI regions, low-resource settings continue to experience an unchecked rise in cases, underscoring the need for urgent, equity-focused interventions. A comprehensive, multidisciplinary approach encompassing prevention, early detection, and treatment is essential to mitigating this emerging public health crisis. Future research should investigate subtype-specific disease burdens, such as differences between endometrioid and serous carcinomas, as well as the potential impact of emerging therapies, including GLP-1 agonists, on cancer incidence.

## Data Availability

The original contributions presented in the study are included in the article/**Supplementary Material**. Further inquiries can be directed to the corresponding author.

## Supplementary material

Supplementary Table S1 The change of uterine cancer or ovarian cancer due to high BMI-related GBD of deaths, YLDs, YLLs and DALYs for different age groups between 1990 and 2021. Abbreviations: ASR, age-standardized rate; YLDs, Years Lived with Disability; YLLs, Years of Life Lost; DALYs, disability-adjusted-life-years.

Supplementary Table S2 The change of uterine cancer or ovarian cancer due to high BMI-related GBD of deaths, YLDs, YLLs and DALYs for different SDI regions between 1990 and 2021. Abbreviations: ASR, age-standardized rate; YLDs, Years Lived with Disability; YLLs, Years of Life Lost; DALYs, disability-adjusted-life-years.

Supplementary Table S3 The change of uterine cancer or ovarian cancer due to high BMI-related GBD of deaths, YLDs, YLLs and DALYs for different GBD regions between 1990 and 2021. Abbreviations: ASR, age-standardized rate; YLDs, Years Lived with Disability; YLLs, Years of Life Lost; DALYs, disability-adjusted-life-years.

Supplementary Table S4 The change of uterine cancer or ovarian cancer due to high BMI-related GBD of deaths, YLDs, YLLs and DALYs for different countries between 1990 and 2021. Abbreviations: ASR, age-standardized rate; YLDs, Years Lived with Disability; YLLs, Years of Life Lost; DALYs, disability-adjusted-life-years.

Supplementary Figure S1 The uterine cancer or ovarian cancer due to high BMI-related numbers and ASRs of deaths, YLDs, YLLs and DALYs for different age groups in 2021. Abbreviations: ASR, age-standardized rate; YLDs, Years Lived with Disability; YLLs, Years of Life Lost; DALYs, disability-adjusted-life-years.

Supplementary Figure S2 The uterine cancer or ovarian cancer due to high BMI-related numbers and ASRs of deaths, YLDs, YLLs and DALYs for different SDI regions in 2021. Abbreviations: ASR, age-standardized rate; YLDs, Years Lived with Disability; YLLs, Years of Life Lost; DALYs, disability-adjusted-life-years.

Supplementary Figure S3 The uterine cancer or ovarian cancer due to high BMI-related numbers and ASRs of deaths, YLDs, YLLs and DALYs for different GBD regions in 2021. Abbreviations: ASR, age-standardized rate; YLDs, Years Lived with Disability; YLLs, Years of Life Lost; DALYs, disability-adjusted-life-years.

Supplementary Figure S4 The uterine cancer or ovarian cancer due to high BMI-related numbers and ASRs of deaths, YLDs, YLLs and DALYs for different countries in 2021. Abbreviations: ASR, age-standardized rate; YLDs, Years Lived with Disability; YLLs, Years of Life Lost; DALYs, disability-adjusted-life-years.
