# Supplementary figures and images for: Global burden of ovarian and uterine cancers attributable to high body-mass index in 204 countries and territories, 1990–2021
Source: Front Oncol. 2025 Oct 20;15:1623926. doi: 10.3389/fonc.2025.1623926 (PMC12580100; doi:10.3389/fonc.2025.1623926)

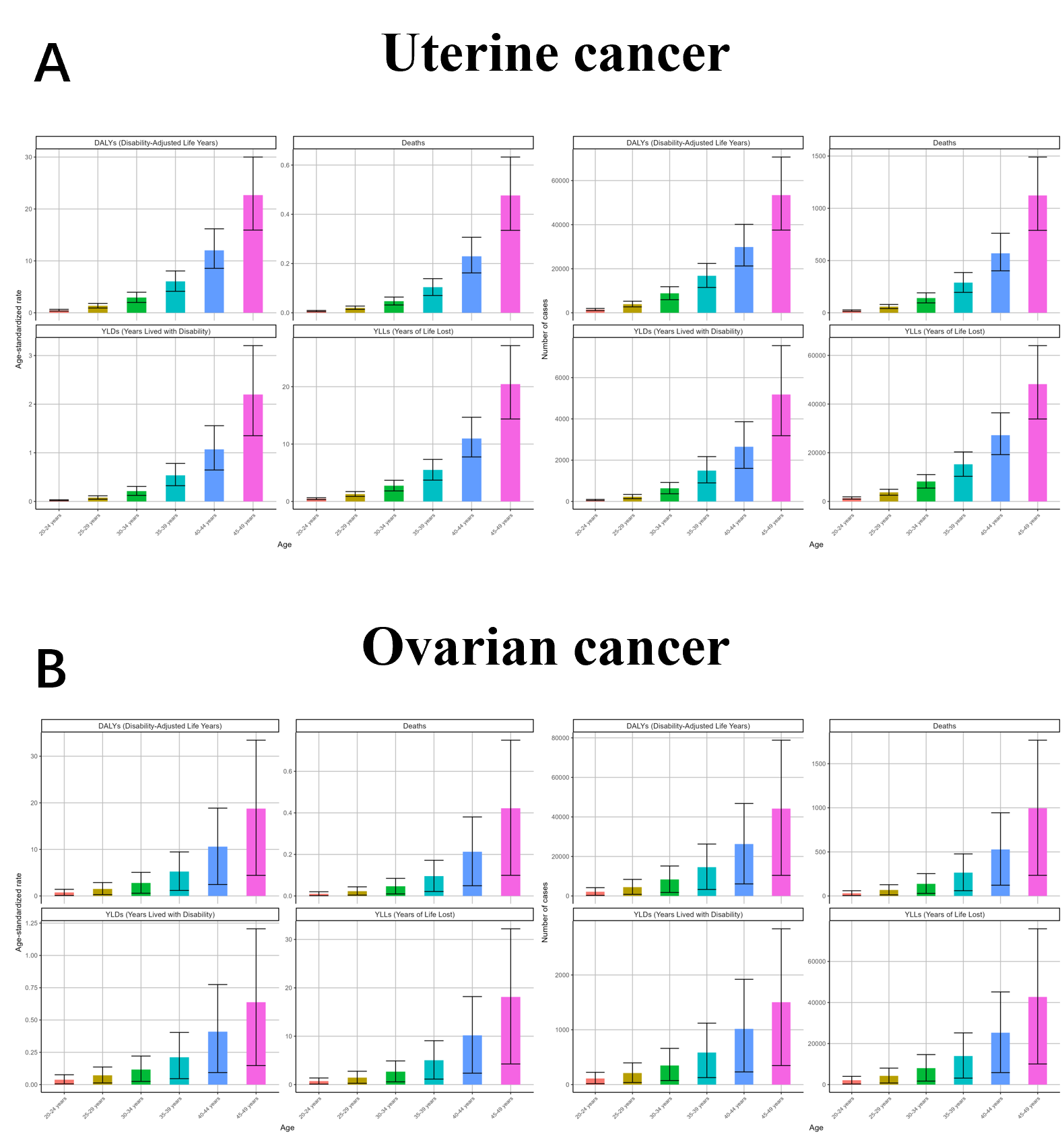

Supplement: Supplementary Figure S1 — The uterine cancer or ovarian cancer due to high BMI-related numbers and ASRs of deaths, YLDs, YLLs and DALYs for different age groups in 2021. Abbreviations: ASR, age-standardized rate; YLDs, Years Lived with Disability; YLLs, Years of Life Lost; DALYs, disability-adjusted-life-years. [file Image1.tif]

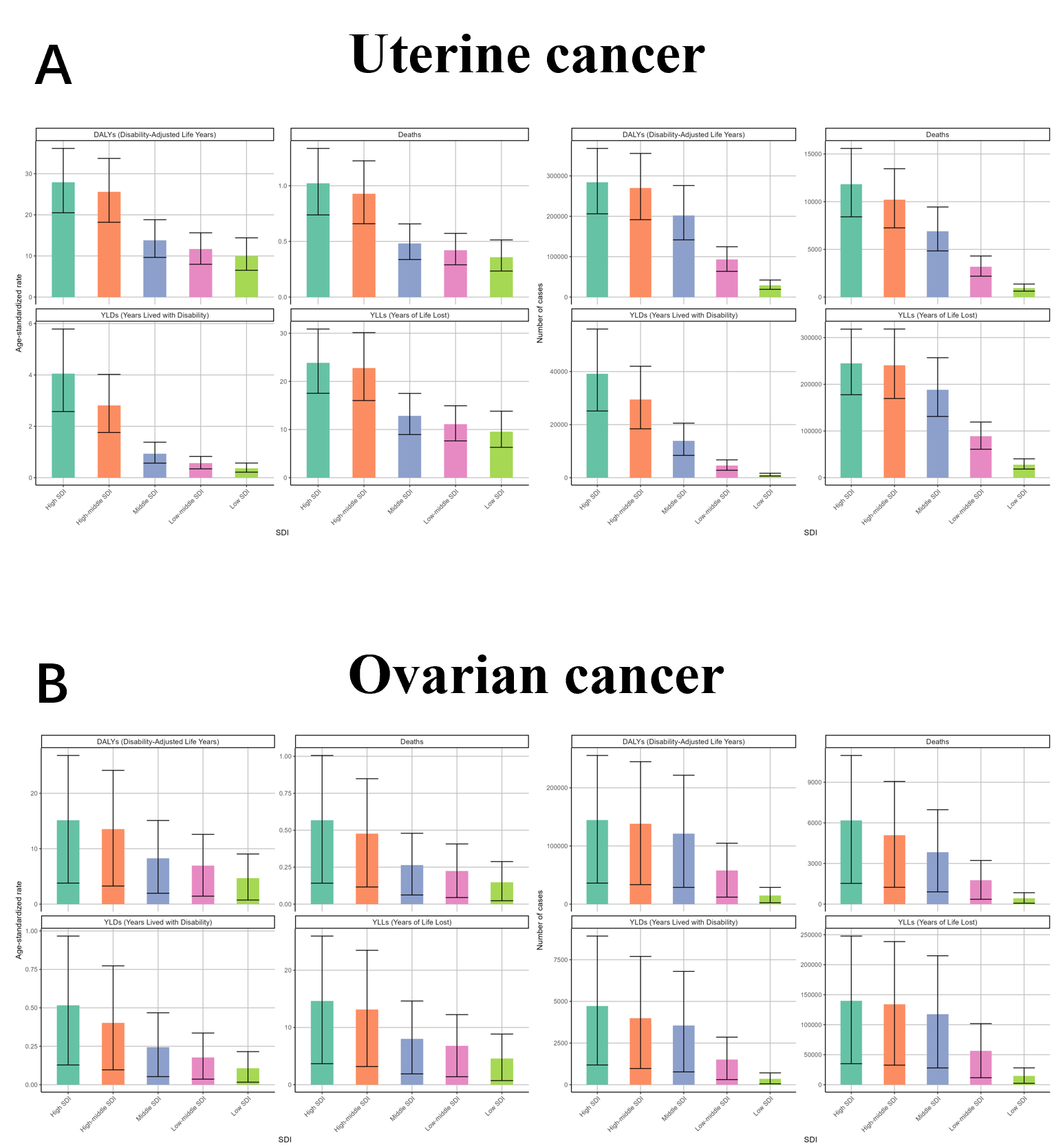

Supplement: Supplementary Figure S2 — The uterine cancer or ovarian cancer due to high BMI-related numbers and ASRs of deaths, YLDs, YLLs and DALYs for different SDI regions in 2021. Abbreviations: ASR, age-standardized rate; YLDs, Years Lived with Disability; YLLs, Years of Life Lost; DALYs, disability-adjusted-life-years. [file Image2.tif]

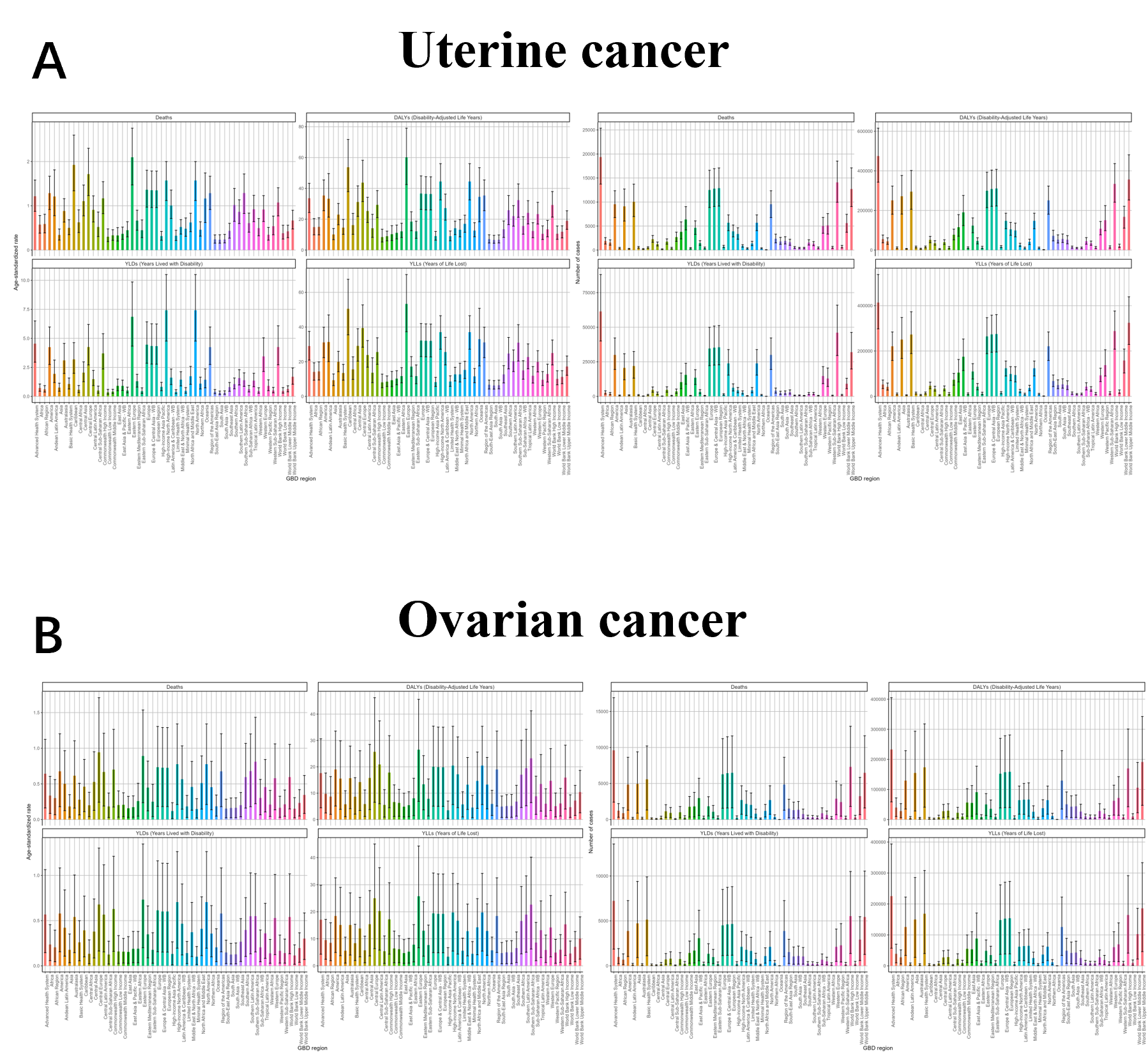

Supplement: Supplementary Figure S3 — The uterine cancer or ovarian cancer due to high BMI-related numbers and ASRs of deaths, YLDs, YLLs and DALYs for different GBD regions in 2021. Abbreviations: ASR, age-standardized rate; YLDs, Years Lived with Disability; YLLs, Years of Life Lost; DALYs, disability-adjusted-life-years. [file Image3.tif]

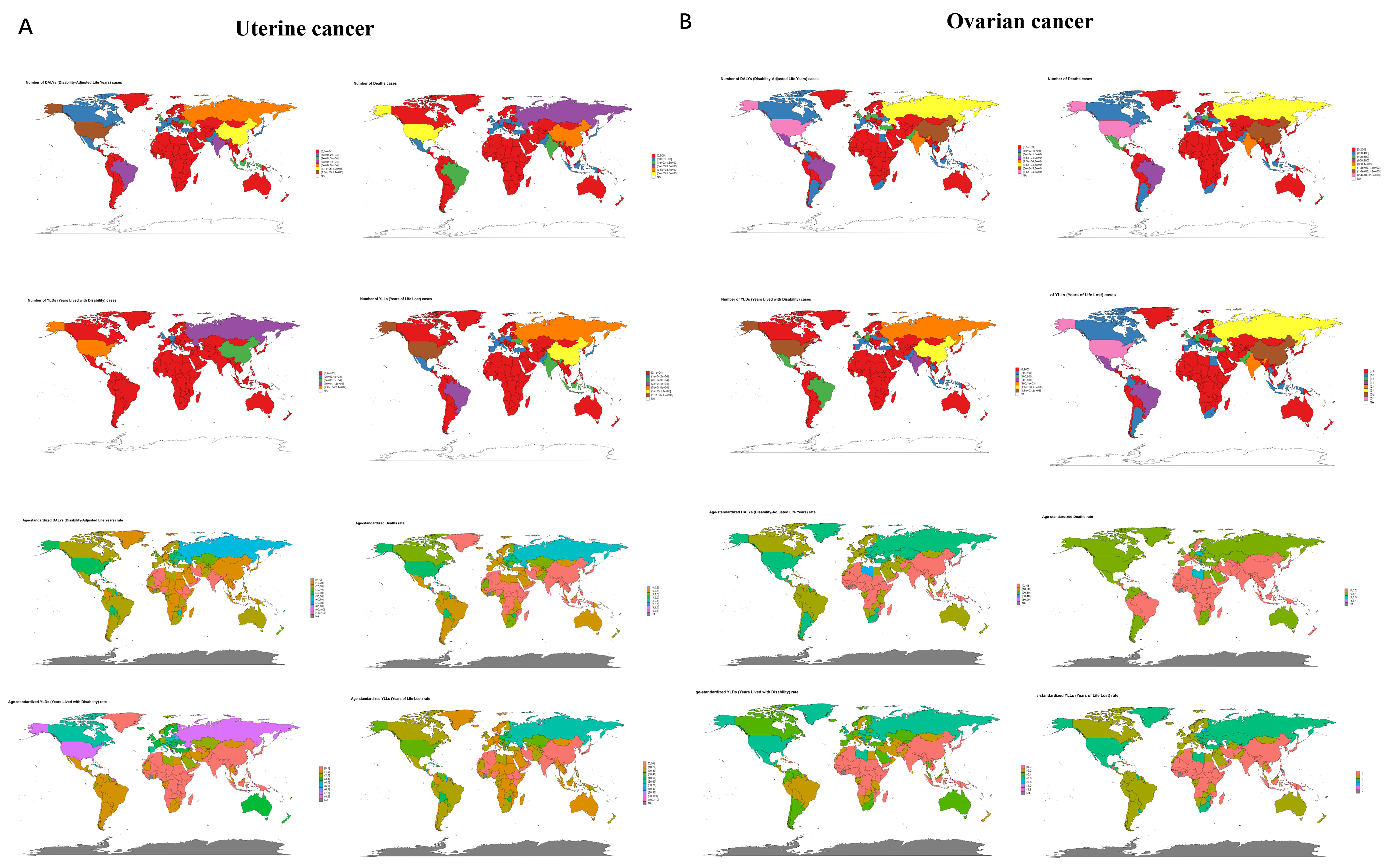

Supplement: Supplementary Figure S4 — The uterine cancer or ovarian cancer due to high BMI-related numbers and ASRs of deaths, YLDs, YLLs and DALYs for different countries in 2021. Abbreviations: ASR, age-standardized rate; YLDs, Years Lived with Disability; YLLs, Years of Life Lost; DALYs, disability-adjusted-life-years. [file Image4.tif]
